# Immunophenotypic Shift From CD8 to CD4 With Anaplastic Lymphoma Kinase (ALK)-Negative Anaplastic Large Cell Transformation in Mycosis Fungoides: A Case Report

**DOI:** 10.7759/cureus.79889

**Published:** 2025-03-01

**Authors:** Timothy H Chan, Ronald Harris, Jagmohan S Sidhu

**Affiliations:** 1 Medicine, State University of New York Upstate Medical University, Syracuse, USA; 2 Medical Oncology, United Health Services, Binghamton, USA; 3 Pathology and Laboratory Medicine, United Health Services, Binghamton, USA

**Keywords:** alk-negative, anaplastic large cell lymphoma, cd4, cd8, cutaneous t cell lymphoma, large cell transformation, mycosis fungoides, phenotypic shift

## Abstract

Mycosis fungoides (MF) classically presents with a CD4+/CD8- immunophenotype at the time of diagnosis. MF may extremely rarely present with CD8+/CD4- and, even more rarely, may shift between CD4+ and CD8+ phenotypes. To date, very few phenotypic shifts in MF have been reported, and just two of them were in the CD8+ to CD4+ direction. Our case report describes another instance of CD8+ to CD4+ phenotypic shift of MF, but with associated transformation to anaplastic lymphoma kinase (ALK)-negative anaplastic large cell lymphoma (ALCL) - the first documented case of its kind. We hope to spread awareness about such rare phenomena to guide future research.

## Introduction

Mycosis fungoides (MF) is the most common subtype of cutaneous T cell lymphoma (CTCL), comprising as much as up to 70% of all cases of CTCL [1,2]. CTCL cells are generally observed to display CD4+/CD8- immunophenotype [1,3,4]. Rarely, CD8+ cytotoxic phenotypes of CTCL may occur. Cytotoxic variants are typically associated with other cytotoxic traits, including CD56+, perforin+, granzyme B+, and TIA-1+ [3]. Specifically for MF in the adult population, only around 5% of MF cases are of the cytotoxic phenotype [5]. Despite some cytotoxic CTCL types being associated with metastasis to extracutaneous sites [3,4], cytotoxic MF is known to have a relatively indolent course [5,6].

Given the low incidence of cytotoxic CTCL, there have naturally been very few documented instances of phenotypic shift in CTCL between CD4+ and CD8+ phenotypes [2,7-12]. Although the shift may occur in either direction, the most common direction is from CD4+/CD8- to CD4-/CD8+. Notably, shifts in the other direction, as well as to CD4+/CD8+ and CD4-/CD8- phenotypes, have been observed, although much more rarely [10]. Bitar et al. estimated that there are fewer than 30 cases in the literature that describe immunophenotypic switch of any kind in peripheral T cell lymphoma (PTCL) [10]. Compared to the relatively indolent nature of MF, these phenotypic switches are generally associated with a worse prognosis [11].

To our knowledge, there have only been two documented cases that describe a shift from the CD4-/CD8+ to CD4+/CD8- direction in PTCL [8,12]. Our case report describes an instance of MF with a phenotypic shift from the CD4-/CD8+ to CD4+/CD8- direction with an associated anaplastic lymphoma kinase (ALK)-negative anaplastic large cell transformation. Given the rarity of phenotypical shift in this direction in CTCL, the following report is the first of its kind to specifically describe this CD8+ to CD4+ immunophenotypic shift in MF with concurrent large cell transformation to ALK-negative anaplastic large cell lymphoma.

## Case presentation

In 2011, a 56-year-old female, with a history of hypertension, diabetes, hyperlipidemia, and hypothyroidism, presented to her dermatologist with increased erythematous, dry, scaly plaques on her upper arms, right abdomen, back, and upper legs. These lesions had been present for 20 years but have recently been progressing significantly. She denied pain, pruritus, and other B symptoms. On physical examination, patches and plaques were found with no lymphadenopathy. Additionally, 4-mm punch biopsies from the right antecubital fossa and right lateral abdomen were taken to rule out clinically suspicious MF. 

**Figure 1:**
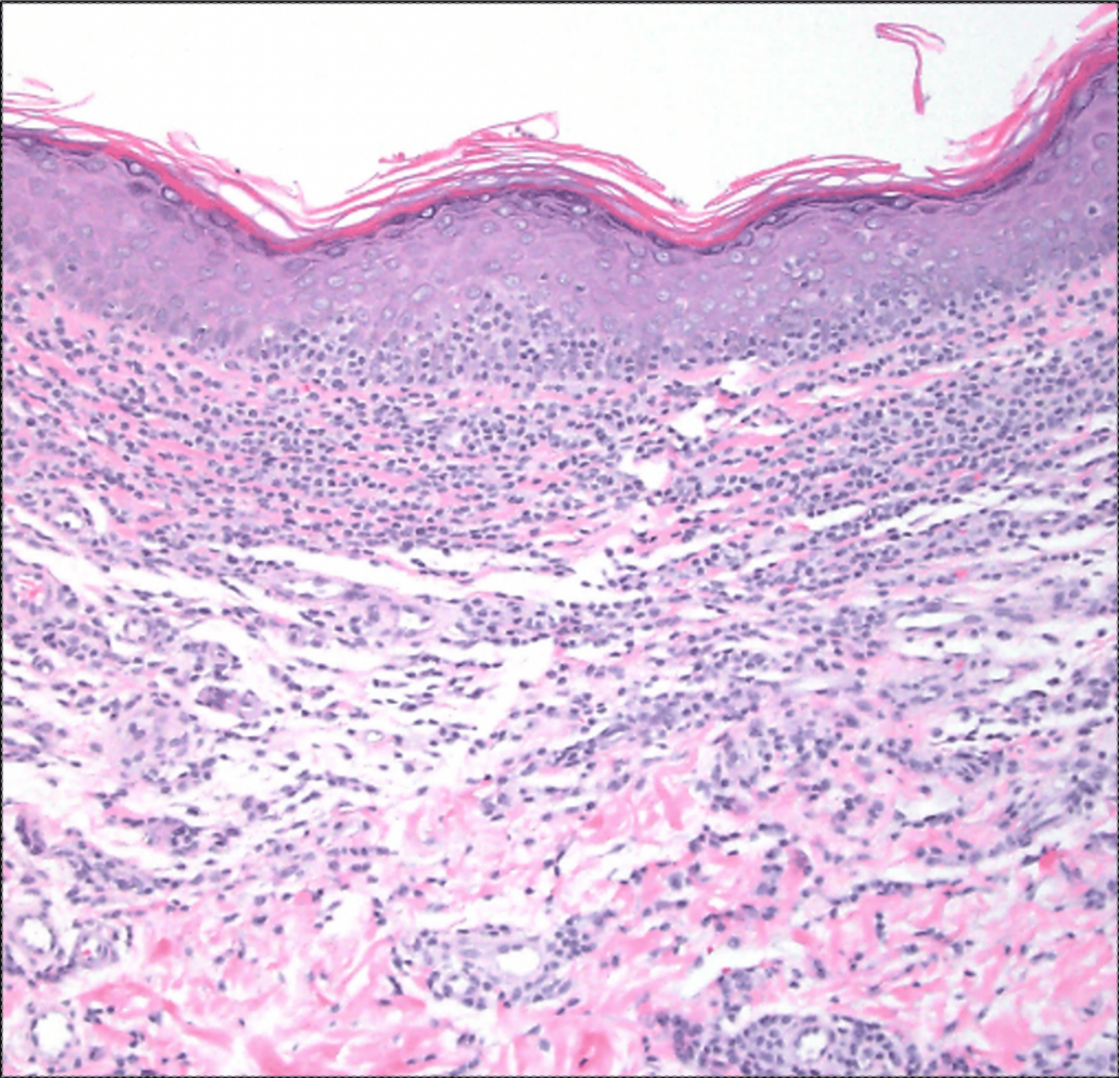
Diffuse infiltrate of small lymphoid cells in the dermis with hugging of and occasional single cell infiltration in the epidermis (H&E x100)

**Figure 2:**
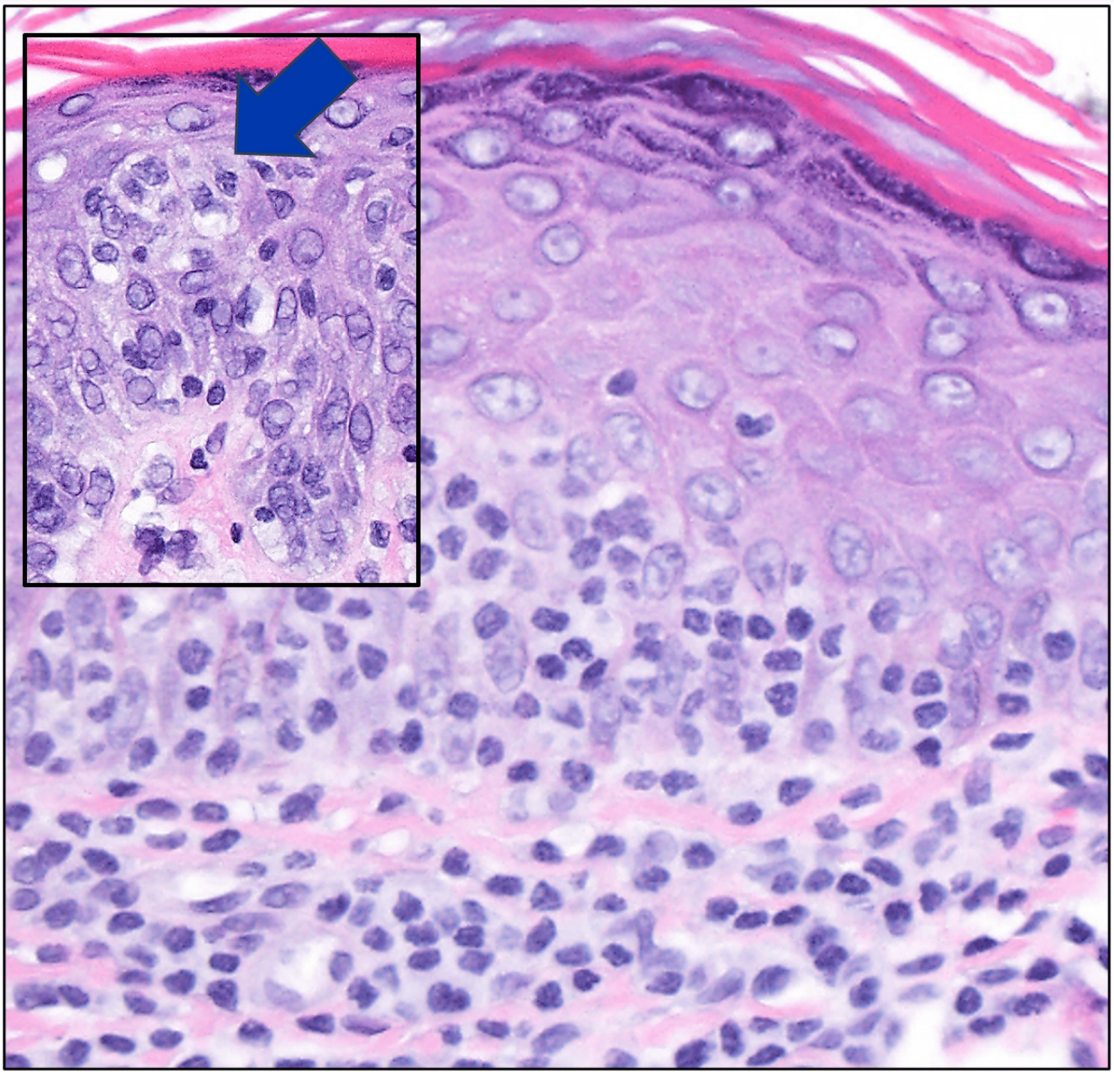
Diffuse intradermal small lymphoid cell infiltrate with hugging of the epidermis and occasional intraepidermal single cell infiltration (H&E x400) An inset (x200) shows a small Pautrier’s microabscess, with a blue arrow pointing toward it and several intraepidermal single lymphocytes.

**Figure 3:**
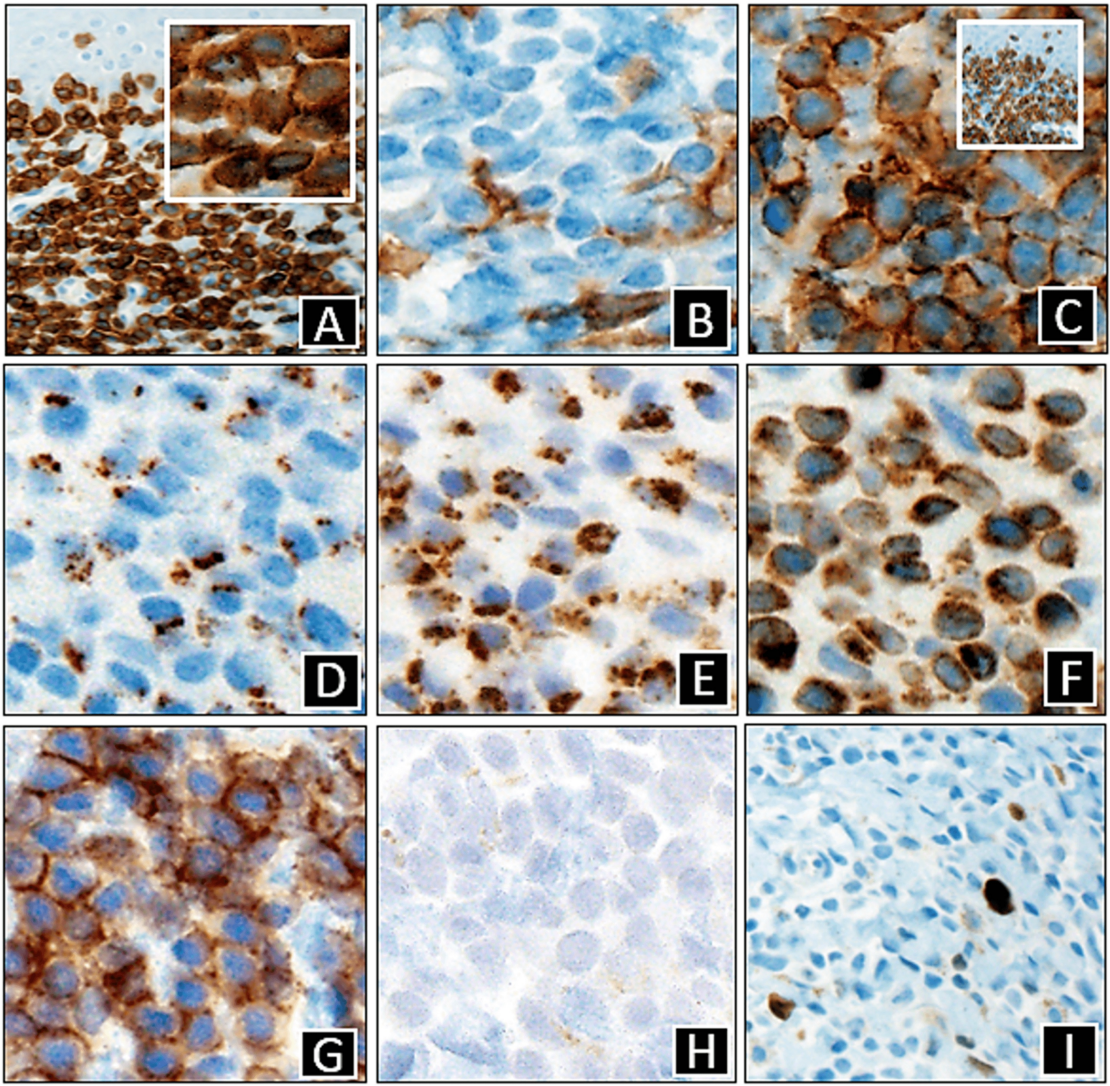
Immunohistochemical stains A. CD3 (X100 with inset at x400); B. CD4 (x400); C. CD8 (X400 with inset at x40); D. Granzyme B (x400); E. TIA-1 (x400); F. Perforin (x400); G. CD56 (x400); H. CD30 (x400); I. Ki67 (x100)

Both sections showed significant lymphoid cell infiltration of the epidermis and dermis (Figures 1-2). The atypical cells were CD2+, CD3+, CD5+, CD8+, CD43+, and CD56+, along with expression of cytotoxic granule proteins, such as perforin, granzyme B, and TIA-1 (Figures 3A, 3C-3G). CD4 stained positively only in some of the T cells and a few normal Langerhans cells (Figure 3B). CD7 stained positively only in some of the T cells. CD30 was negative (Figure 3H). Both specimens demonstrated Ki-67 staining only in some of the cells, with a Ki67 labeling index of about 1% (Figure 3I). The lack of CD7 expression in the atypical lymphocytes suggested a T-cell lymphoma, and the presence of Pautrier microabscesses narrowed the differential diagnosis to MF (Figure 2), consistent with her clinical presentation. Co-expression of CD8, CD56, and cytotoxic granule proteins suggested the diagnosis of a cytotoxic variant of MF, and the presence of T-cell clonality via polymerase chain reaction (PCR) supported this diagnosis. 

The patient was started on five cycles of CHOP (cyclophosphamide, doxorubicin, vincristine, prednisone) chemotherapy. However, her skin lesions did not improve, so her lymphoma was assumed to be clinically refractory to this regimen. She was switched to Folotyn (pralatrexate) but discontinued after receiving three injections due to the development of painful oral mucositis and poor treatment tolerance. The patient was switched to Istodax (romidepsin) for 32 cycles and noticed some clinical improvement. However, the lesions continued to persist. In 2014, her regimen was modified to romidepsin with concurrent PUVA phototherapy, which she tolerated well. Around this time, another shave biopsy was performed on her right flank, which confirmed cytotoxic CD4-/CD8+ MF. She continued with the romidepsin/phototherapy regimen for years, receiving romidepsin every two or three weeks and psoralen plus ultraviolet A (PUVA) phototherapy treatment intermittently around two or three times per week. When receiving romidepsin every three weeks, her lesions would begin to flare the week before her infusion; however, it was difficult to manage her schedule with receiving romidepsin every two weeks. She was given topical clobetasol cream to use as needed for these flares. Her MF remained clinically stable on this regimen for years, and a positron emission tomography (PET) scan performed in 2023 confirmed no significant activity regarding her lymphoma. However, our patient noticed her lesions spreading distally on her extremities in 2024. A 2.3 x 0.8 x 0.2 cm excisional biopsy from her left anterior shoulder lesion was taken in response to the clinical progression of her MF.

**Figure 4:**
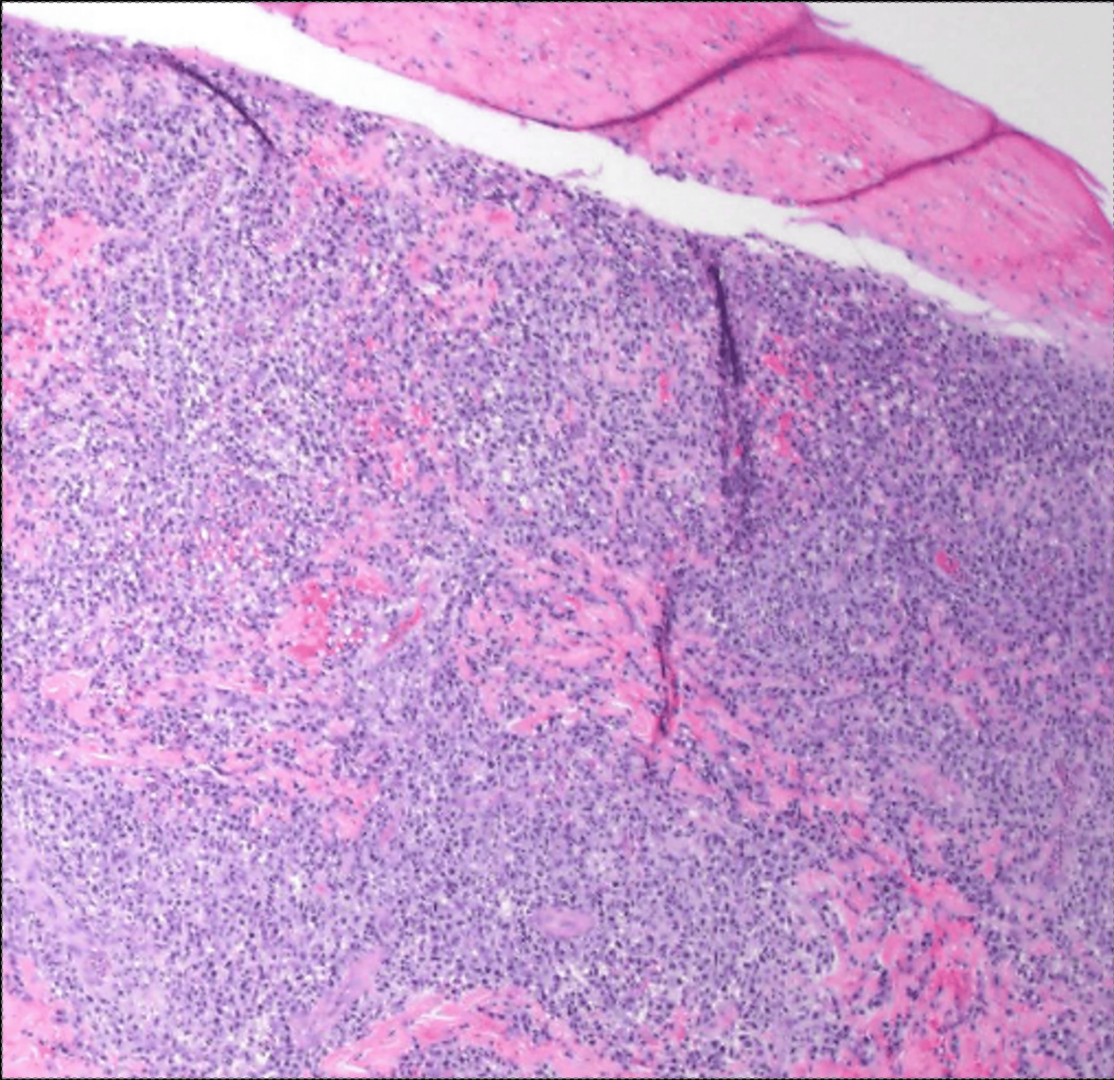
Diffuse and dense infiltrate of lymphoid cells in the dermis with necrosis of overlying epidermis (H&E x40)

**Figure 5:**
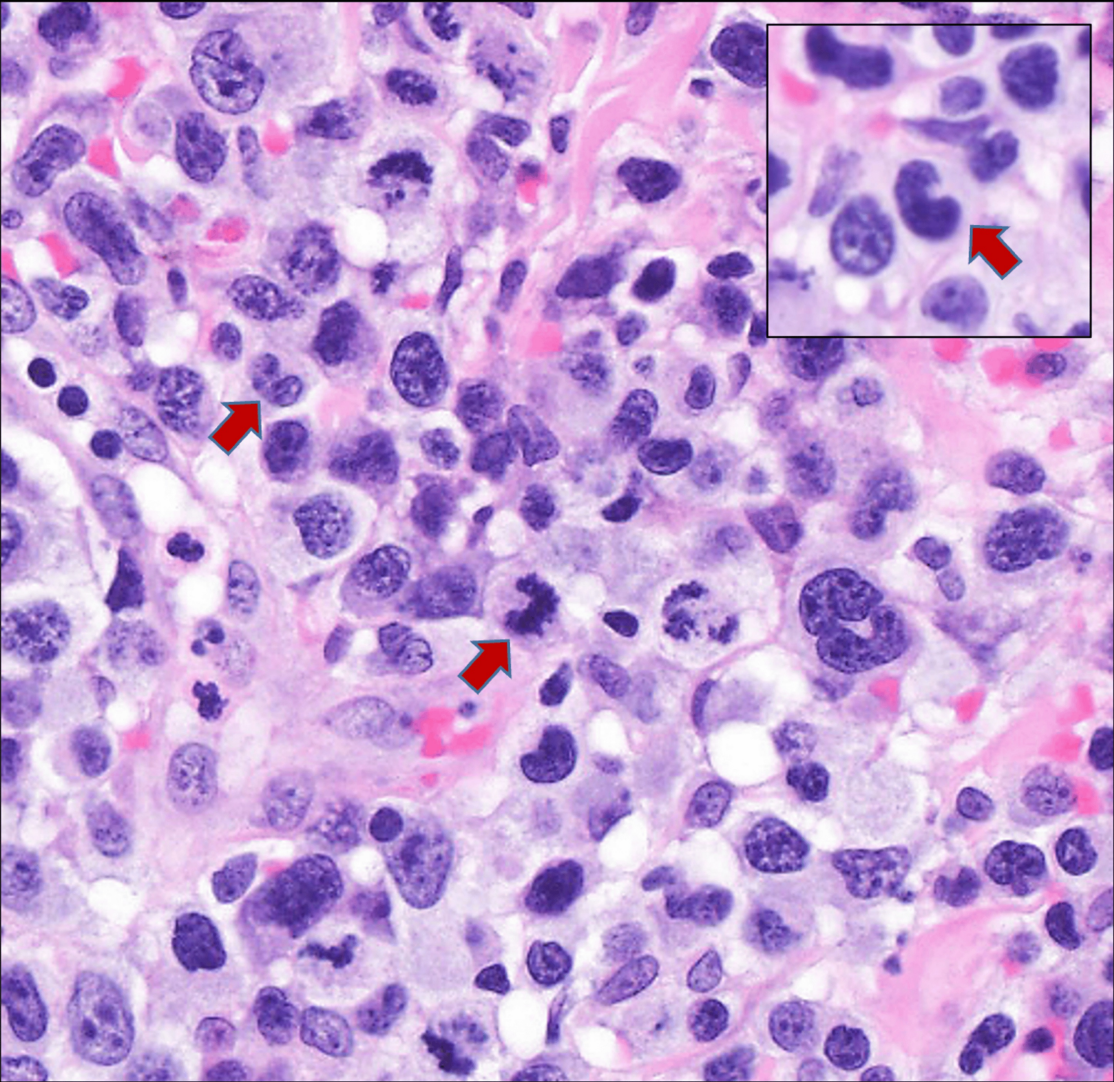
Diffuse and dense infiltrate of large anaplastic lymphoid cells in the dermis with numerous mitoses (H&E x400) Red arrows in the main picture and in the inset show “hallmark” cells.

**Figure 6:**
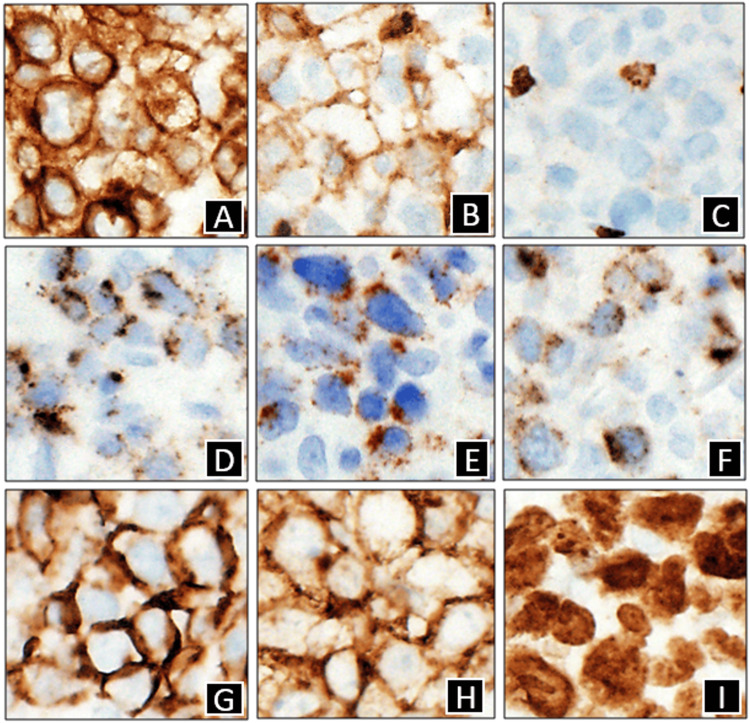
Immunohistochemical stains x400 A. CD3; B. CD4; C. CD8; D. Granzyme B; E. TIA-1; F. Perforin; G. CD56; H. CD30; I. Ki67

**Figure 7:**
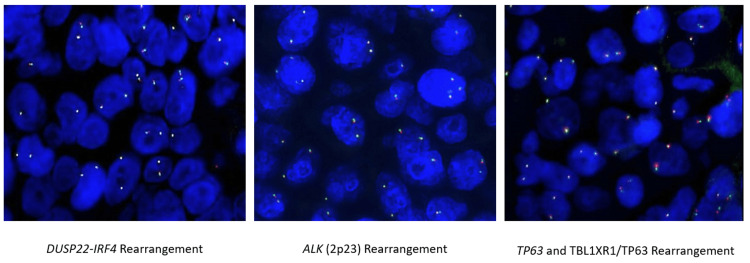
Negative FISH for the DUSP22-IRF4 rearrangement, ALK (2p23) rearrangement, and TP63 rearrangement ALK: anaplastic lymphoma kinase; FISH: fluorescence in situ hybridization

The specimen showed diffuse, large cell infiltrates in the dermis with epidermotropism (Figures 4, 5). The large cells showed a moderate/high nuclear to cytoplasmic ratio with prominent nucleoli. Occasional “hallmark” cells were seen (Figure 5). No “doughnut” cells were present. The neoplastic cells weakly expressed CD4+, and CD8 was negative (Figures 6B, 6C). These cells also expressed cytotoxic markers such as CD56+ and perforin, granzyme B, and TIA-1 (Figures 6D-6G). The large cells expressed pan-T markers, such as CD2, CD3 (Figure 6A), and Bcl-2, which confirmed our suspicion that the large cell transformation was of T-cell immunophenotype. Other positive stains were CD25, CD30 (Figure 6H), CD45, and TCRβF1. The large cells did not express CD5, CD7, CD8, TCRdelta, ALK1, S100, and CD1a. A Ki67 labeling index was very high at around 90% (Figure 6I). p63 was also only focally positive in the large cells. The lesional infiltrate also hosted a few small cells with wrinkled nuclei that expressed CD8+, CD5+, CD7+, CD56+, and cytotoxic granule proteins. Later fluorescence in situ hybridization (FISH) studies ruled out the rearrangement of ALK, DUSP22, and TP63 in this lymphoma (Figure 7). Given the patient’s prior history, she was diagnosed with ALK-negative anaplastic large-cell transformation in a cytotoxic variant of MF.

Given the rarity of her case, our patient was referred to a T-cell lymphoma specialist at a large academic tertiary center. Her romidepsin regimen was switched to weekly brentuximab infusions, a CD30-directed monoclonal antibody that is used to treat anaplastic large-cell lymphoma by inducing apoptosis in CD30+ cells [13]. Her treatment is still ongoing at this time. Table 1 summarizes the histology and management of her lymphoma before and after the shift/transformation.

**Table 1:** Summary of findings and associated treatments by year of presentation

Year of Presentation	Histological Characteristics	Treatment
2011	CD4-, CD8+, perforin+, granyzme B+, TIA-1+, CD56+, CD30-	CHOP x 5 cycles, then romidepsin + PUVA phototherapy
2024	CD4+, CD8-, perforin+, granyzme B+, TIA-1+, CD56+, CD30+. Large cell transformation without ALK, DUSP22, and TP63 rearrangement	Brentuximab

## Discussion

Given its rarity in the literature, our immediate interest was in the observed CD4-/CD8+ to CD4+/CD8- immunophenotypic shift. As mentioned previously, we believe that there have only been two previously documented cases of a shift in this direction to date. Aung et al. described a case in 1974 of a 41-year-old male with PTCL who exhibited a shift from a CD3+/CD4-/CD8+ phenotype to a predominant CD4+ phenotype over the course of nine years [8]. The second documented case of a phenotypic shift in this direction is described by Al-Ibraheemi et al. of a 40-year-old female with PTCL who demonstrated a shift from CD4-/CD8+/TIA1+ to CD4+/CD8-/TIA1- [12]. Notably, both cases had extracutaneous involvement at some point during its course (of the lung and nasal septum, respectively) and demonstrated considerable resistance to chemotherapy regimens. The pathophysiology behind immunophenotypic shifts is uncertain; given that these shifts frequently occur after the initiation of chemotherapy, proposed mechanisms include therapy-induced antigen changes or preferential selection of certain subclones with survival advantages in response to therapy [10,11].

The malignant large cells found in the 2024 specimen notably retained several of their cytotoxic markers from 13 years earlier, including expression of CD56+ and cytotoxic granule proteins (perforin, granzyme B, and TIA-1). The retention of these markers (despite switching from CD8+ to CD4+) supports our belief that the large cells are indeed a product of a phenotypical shift from the prior lymphoma rather than a de novo presentation.

Morphological large-cell transformation in MF has been recorded before, with literature suggesting this happening in as much as 50% of cases of advanced MF [14,15]. Primary cutaneous large cell lymphomas are typically found with the CD30+ immunophenotype [11,14], which was gained in our case between the 2011 and 2024 specimens. Large cell transformation of CTCL also is associated with the loss of pan T-cell antigens (such as CD7) before and during transformation [14], also consistent with our case. The clinical implications of this are also mixed; conventionally, large-cell transformation in MF was thought to be associated with increased tumor stage progression and, therefore, a worse prognosis [14]. However, this worse prognosis might only be associated with several particular histopathological features [15].

In the context of our observed large cell transformation, we established the diagnosis as one of the ALK-negative anaplastic large cell lymphoma (ALCL) subtypes. ALCLs are considered CD30+ T cell lymphomas that can be broadly subcategorized as either ALK-positive or negative [16-18]. As consistent with our case, ALK-negative ALCL typically expresses cytotoxic traits [19], with the exception of variants that present with DUSP22 rearrangements [16]. DUSP22 rearrangements can be seen in up to 30% of ALK-negative ALCL via a t(6;7) translocation, and they are generally associated with an improved prognosis [16-18]. Conversely, TP63 rearrangements can also be observed in ALK-negative ALCL via inv(3)(q26q28) chromosomal inversion, increasing expression of the p63 protein, which may explain its association with worse prognosis [16-18]. The possibility of having an ALK-negative ALCL that also lacks both DUSP22 and TP63 rearrangements, such as ours, is also possible; this “triple-negative” variant is described in approximately 80% of ALK-negative ALCLs [20]. Triple-negative ALCL has been found to generally be refractory to treatment with relatively poor five-year survival rates [20]. To our knowledge, there does not appear to be empirical literature about triple-negative ALCL transformed from MF, much less from a cytotoxic variant of MF, so it is difficult to confidently make predictions about the clinical course for our patient. However, morphological large-cell transformation and triple-negative ALCL both independently suggest a difficult clinical course.

To our knowledge, the only context in which an immunotypic shift in CTCL has been seen concurrently with any sort of large cell transformation was described by Bitar et a., in which a 78-year-old male with MF showed a phenotypic shift from CD4+/CD8- to double-negative CD4-/CD8- with large cell transformation [10]. This reinforces our claim that our case is the first documented instance of MF shift from CD4-/CD8+ to CD4+/CD8- with an associated ALK-negative ALCL transformation.

## Conclusions

We reported a rare case of a phenotypic shift from the CD8+ to CD4+ direction in MF with concurrent ALK-negative ALCL transformation, the first of its kind to be documented in the literature. The implications of both the CD8+ to CD4+ phenotypic shift and triple-negative ALCL independently suggest a comparatively poor prognosis. Given the rarity and variability in both immunophenotypic shifts and ALCL transformation, we hope that this case contributes to the continuously evolving dialogue surrounding both phenomena.
